# Prediction Model of Lymph Node Metastasis in Cervical Cancer Based on MRI Habitat Radiomics

**DOI:** 10.3390/cancers18010152

**Published:** 2025-12-31

**Authors:** Mei Wang, Yu Cao, Weiwei Zhang, Yun Liang, Jizhao Liu, Junqiang Lei

**Affiliations:** 1 The First Clinical Medical College, Lanzhou University, Lanzhou 730000, China; wangmeilz@163.com (M.W.); 885200930@qq.com (Y.C.); 19513575835@163.com (Y.L.); 2 Department of Obstetrics and Gynecology, The First Hospital of Lanzhou University, Lanzhou 730000, China; 3 Gansu Provincial Clinical Research Center for Gynecological Oncology, Lanzhou 730000, China; 4 The First Clinical Medical College of Gansu University of Traditional Chinese Medicine, Lanzhou 730000, China; ww.zhang@gszy.edu.cn; 5 School of Information Science and Engineering, Lanzhou University, Lanzhou 730000, China; liujz@lzu.edu.cn; 6 Gansu Province Clinical Research Renter for Radiology Imaging, The First Hospital of Lanzhou University, Lanzhou 730000, China; 7 Intelligent Imaging Medical Engineering Research Center of Gansu Province, The First Hospital of Lanzhou University, Lanzhou 730000, China

**Keywords:** cervical cancer, radiomics, habitat radiomics, machine learning, feature engineering

## Abstract

**Background**: Radiomics provides a non-invasive approach for predicting lymph node metastasis (LNM) in cervical cancer, but conventional whole-tumor analysis often overlooks intratumoral heterogeneity. **Methods**: This study aimed to develop and validate an MRI-based habitat radiomics model for preoperative prediction of pelvic LNM in early-stage cervical cancer. Tumor regions were delineated on diffusion-weighted imaging, and intratumoral habitats were generated using unsupervised K-means clustering. Radiomic features were extracted from whole tumors and habitat subregions, combined with clinical variables, and selected using correlation analysis and LASSO regression. Four models—clinical, conventional radiomics, habitat radiomics, and combined—were constructed and evaluated. **Results**: In internal validation, the combined model achieved the best performance (AUC = 0.895), outperforming the clinical (AUC = 0.799), conventional radiomics (AUC = 0.611), and habitat models (AUC = 0.872). Calibration and decision curve analyses demonstrated good agreement and clinical utility. **Conclusions**: Integrating habitat-based radiomics with clinical factors significantly improves the preoperative prediction of LNM, providing a robust and clinically applicable tool for individualized management of cervical cancer patients.

## Simple Summary

Lymph node metastasis is an important factor affecting treatment decisions and prognosis in patients with cervical cancer, but it is difficult to accurately assess before surgery using conventional imaging methods. In this study, we developed a new prediction model based on magnetic resonance imaging (MRI) radiomics that takes tumor heterogeneity into account. By dividing tumors into different intratumoral subregions (habitats) and combining imaging features with clinical information, we were able to more accurately predict pelvic lymph node metastasis in patients with early-stage cervical cancer. Our results show that this habitat-based radiomics model performs better than traditional clinical or whole-tumor radiomics models and may help clinicians better plan individualized treatment strategies before surgery.

## 1. Introduction

In cervical cancer, lymph node metastasis (LNM) is one of the most critical prognostic factors, directly influencing treatment decisions, survival outcomes, and individualized patient management [1]. Current clinical evaluation largely relies on preoperative imaging and intraoperative pathology; however, the sensitivity of MRI for detecting nodal involvement remains limited (approximately 30–50%), which may result in overtreatment or undertreatment [2,3]. Radiomics, as an emerging imaging analysis technology, has been increasingly applied in disease diagnosis, prognostic evaluation, and personalized treatment strategies by extracting high-throughput features to quantitatively characterize tumor phenotypes [4,5]. By providing quantitative imaging biomarkers, radiomics offers a promising non-invasive approach to overcome these limitations [6]. However, conventional radiomics typically regards tumors as homogeneous entities, thereby neglecting intratumoral heterogeneity, which limits predictive accuracy and robustness in clinical practice. To address this limitation, we introduce the concept of tumor habitat and divide tumors into three-dimensional pixel-level subregions to better capture spatial heterogeneity and reflect distinct biological behaviors. Previous studies have demonstrated the additional predictive value of habitat-based analysis in cancers such as breast cancer and glioma [7,8,9]. On this basis, we systematically applied MRI-based habitat radiomics to the prediction of cervical cancer LNM for the first time and compared it with conventional radiomics and clinical models, aiming to develop a more accurate and clinically applicable prediction tool.

This study offers three major contributions:

1. Development of a habitat radiomics framework for cervical cancer. By applying unsupervised K-means clustering to preoperative $T_{2}$-weighted images ($T_{2}$WI) and diffusion-weighted images (DWI), we generated intratumoral subregions within the region of interest (ROI). These subregions capture spatial heterogeneity that cannot be represented by whole-tumor radiomics, thereby providing complementary biomarkers for LNM prediction [10,11];
2. Robust feature extraction and selection. Following IBSI guidelines [12], we extracted more than 1000 features, including morphological descriptors, first-order statistics, and texture metrics. After Z-score normalization, redundancy was reduced through correlation analysis, and a stable subset of features was selected using least absolute shrinkage and selection operator (LASSO) regression [13]. These features demonstrated high reproducibility and robustness for model construction;
3. Construction and validation of prediction models. We developed and validated four models, including clinical models, conventional radiomics models, habitat radiomics models, and combined models. In the internal independent validation cohort, the AUCs of the four models were 0.799, 0.611, 0.872, and 0.895, respectively. Among them, the combined model achieved the best overall performance, and calibration and decision curve analyses confirmed its reliability and clinical utility. The nomogram generated by the model may reduce unnecessary surgical exploration, enable accurate individualized risk stratification, and optimize preoperative management strategies for cervical cancer [7,14,15,16,17].

The remainder of this paper is organized as follows: Section 2 describes the materials and methods, Section 3 presents the results, Section 4 provides the discussion, and Section 5 concludes the study.

## 2. Materials and Methods

### 2.1. Patient Cohort

#### 2.1.1. Study Design and Ethical Approval

This was a single-centre retrospective study conducted in the Department of Obstetrics and Gynecology at the First Hospital of Lanzhou University [18]. The study protocol was approved by the institutional review board, and the requirement for informed consent was waived owing to the use of anonymized data.

#### 2.1.2. Inclusion and Exclusion Criteria

We consecutively enrolled patients with pathologically confirmed early-stage cervical cancer who underwent preoperative pelvic MRI between January 2017 and December 2022.

The inclusion criteria were as follows:

1. Histopathological confirmation of cervical cancer;
2. Availability of complete MRI data acquired within two weeks before surgery, including $T_{2}$WI, DWI, and ADC sequences;
3. Surgical treatment comprising lymphadenectomy with pathological assessment of nodal status;
4. Complete clinicopathological records.

The exclusion criteria were as follows:

1. History of prior pelvic chemoradiotherapy or systemic therapy, or evidence of extensive distant metastases;
2. Poor image quality or incomplete MRI sequences;
3. Incomplete clinical data;
4. Absence of surgically confirmed nodal status.

A total of 149 patients met the inclusion criteria and were included in the final analysis [10]. The median age was 51 years (range, 28–70 years). All MRI examinations were performed using the same scanner model, and the entire dataset adopted unified acquisition parameters and strict quality control. The baseline characteristics of the 149 enrolled patients are shown in Table 1 [19].

### 2.2. MRI Acquisition and Segmentation

#### 2.2.1. Workflow

**Figure 1:**
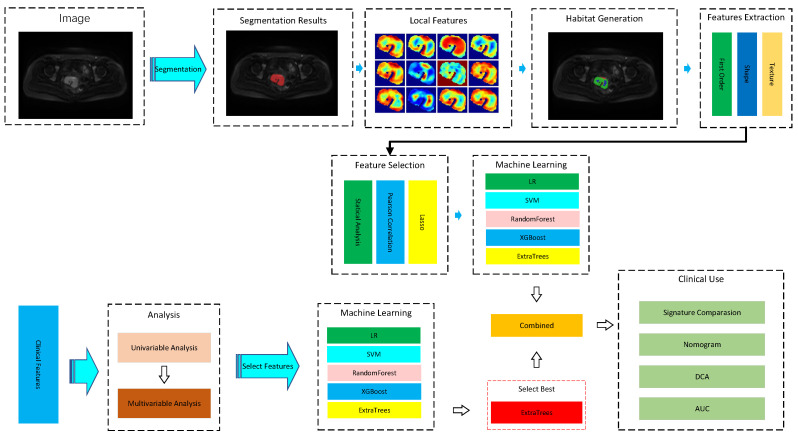
Overall workflow of this study.

The overall workflow of this study consisted of four major steps: image segmentation, feature extraction, feature selection, and model construction with validation. In the preprocessing stage, tumor regions were precisely delineated through manual segmentation to ensure accurate localization for subsequent analysis. A comprehensive set of radiomic features was then extracted, including first-order statistics, morphological descriptors, texture-based metrics, as well as local and habitat features, which were further combined with clinical variables. Feature selection was performed using a multi-step strategy to reduce dimensionality and redundancy: Pearson correlation analysis was applied to eliminate highly correlated features, LASSO regression was employed for sparse selection, and ExtraTrees was used to evaluate feature importance, thereby retaining the most informative predictors. The selected features were then fed into multiple machine-learning classifiers, including Random Forest, XGBoost (v3.0.0), ExtraTrees, Support Vector Machine, and Logistic Regression, to construct predictive models with cross-validation to ensure robustness and generalizability. Subsequently, univariable and multivariable analyses were conducted to assess the independent predictive value of both radiomic and clinical features, and a nomogram was developed to achieve individualized risk estimation. Finally, model performance was systematically evaluated by comparing different feature signatures and by applying decision curve analysis (DCA) and the area under the receiver operating characteristic curve (AUC), which enabled the assessment of discrimination, clinical utility, and net benefit. The threshold probability range of 0.1–0.4 was selected for DCA because this interval reflects clinically relevant decision thresholds in the preoperative management of early-stage cervical cancer. A predicted probability below 0.1 rarely alters surgical planning, whereas probabilities above 0.4 typically prompt more aggressive nodal assessment. This range is therefore consistent with real-world clinical decision-making and adheres to CLAIM recommendations. The overall study workflow is illustrated in Figure 1.

#### 2.2.2. MRI Acquisition and ROI Segmentation

We used unified parameters under a standardized scheme to acquire pelvic MRI on the same Siemens scanner model. Imaging protocols included axial $T_{2}$-weighted imaging ($T_{2}$WI), diffusion-weighted imaging (DWI), and apparent diffusion coefficient (ADC) maps for tumor delineation and cross-checking [20]. The region of interest (ROI) was manually segmented on the DWI maps and cross-checked with $T_{2}$WI and ADC images using ITK-SNAP software (v4.2.2). The delineation was completed independently by two radiologists with more than 10 years of gynecological imaging experience. Reproducibility between observers was assessed in 30 randomly selected cases, with one observer repeating the segmentation after a two-week interval. Any discrepancies were resolved by consensus [21]. Manual delineation on DWI ensured accurate tumor localization and minimized the inclusion of surrounding non-tumoral tissues and background noise, providing a high-quality ROI for subsequent analysis.

### 2.3. Image Preprocessing

To ensure consistency of the input data and enhance the generalizability of the prediction model, all MRI images were preprocessed prior to feature extraction. First, images were resampled to an isotropic voxel size of $1\times 1\times 1\;{\mathrm{mm}}^{3}$ to maintain spatial consistency between patients [22]. In addition, N4 bias field correction was applied to mitigate intensity inhomogeneity inherent to MRI acquisition, thereby reducing low-frequency intensity non-uniformity and improving voxel-level signal reliability. Second, intensity normalization combined with Z-score standardization was applied to reduce gray-level variability caused by scanner heterogeneity and acquisition parameter differences [6]. These preprocessing steps collectively act to suppress technical noise and improve the robustness of downstream radiomic analysis. Finally, random data augmentation was performed to increase training diversity and improve model robustness [23]. Augmentation strategies included random rotation within ±15°, horizontal and vertical flipping, scaling in the range of 0.9–1.1, and the addition of Gaussian noise (σ=0.01) to enhance noise tolerance [24,25].

### 2.4. Radiomic Feature Extraction and Selection

#### 2.4.1. Tumor Habitat Segmentation

**Figure 2:**
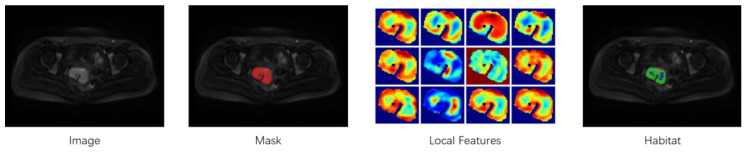
Workflow of tumor habitat segmentation and feature extraction.

On the basis of manually delineated ROIs, unsupervised k-means clustering (*k* = 2∼10) was applied at the voxel level to capture spatial heterogeneity within tumors. The Calinski–Harabasz index, Silhouette coefficient, and Davies–Bouldin index were used to determine the optimal number of clusters, resulting in the identification of three stable subregions (habitats) [26,27,28,29]. The segmentation scheme was used for subsequent analyses. The workflow is shown in Figure 2, including the original MRI image, the tumor mask, the three-dimensional pixel-level feature map, and the resulting subregion division.

It should be noted that the purpose of unsupervised K-means clustering in this study was not noise removal in the conventional image-processing sense. Instead, clustering was applied exclusively to preprocessed voxel data within the manually defined tumor ROI to explore intratumoral signal heterogeneity and identify biologically meaningful subregions (habitats). All input voxels for clustering had already undergone quality control through prior preprocessing and ROI restriction.

#### 2.4.2. Feature Extraction

According to the Image Biomarker Standardisation Initiative (IBSI) guidelines, radiomic features were extracted from ROIs and habitat subregions. The PyRadiomics package (v3.0.1) was used to calculate a comprehensive set of features, including:

1. First-order statistics (such as mean, median, skewness, and kurtosis);
2. Morphological characteristics (e.g., sphericity and compactness);
3. Texture features derived from the gray-level co-occurrence matrix (GLCM), gray-level run-length matrix (GLRLM), gray-level size zone matrix (GLSZM), and neighborhood gray tone difference matrix (NGTDM) [30].

#### 2.4.3. Feature Reproducibility Assessment

To ensure feature stability, intra- and inter-observer reproducibility was evaluated in 30 randomly selected cases. Two experienced radiologists independently delineated ROIs and calculated intraclass correlation coefficients (ICC). Features with an ICC ≥ 0.80 were considered reproducible and retained for subsequent modeling [12]. In the framework proposed by McGraw and Wong, a two-way random-effects model with absolute agreement [ICC(2,*k*)] was adopted. The formula is given as follows:

(1) $$ICC\left(2,k\right)=\frac{MS_{R}-MS_{E}}{MS_{R}+\left(k-1\right)MS_{E}+\frac{k}{n}\left(MS_{C}-MS_{E}\right)}.$$

Here, $MS_{R}$ represents the mean square for rows, $MS_{C}$ represents the mean square for columns, and $MS_{E}$ represents the residual error. In addition, ICC(2,1) was used to evaluate inter-observer consistency of manual segmentation. ICC values below 0.5 were interpreted as poor reliability, values between 0.5 and 0.75 as moderate reliability, values between 0.75 and 0.9 as good reliability, and values above 0.9 as excellent reliability.

### 2.5. Feature Selection and Model Construction

Feature selection was performed using a three-step procedure. First, univariate logistic regression was used to identify features significantly associated with nodal status (p<0.05). Second, pairwise correlation analysis was applied to remove highly collinear variables (|*r*| > 0.9). Finally, the least absolute shrinkage and selection operator (LASSO) regression with ten-fold cross-validation was implemented to determine the optimal regularization parameter λ. As λ increased, coefficients of less informative features progressively shrank toward zero, leaving only the most predictive variables. At the optimal λ, nine robust radiomic features were retained, encompassing first-order statistics, texture descriptors derived from GLCM and GLDM, and higher-order wavelet features. Together, these features provided complementary representations of tumor heterogeneity and formed the basis for subsequent model development.

In the subsequent phase of analysis, four representative machine-learning classifiers—namely logistic regression (LR), support vector machine (SVM), random forest (RF), and extreme gradient boosting (XGBoost)—were systematically constructed and optimized. To comprehensively evaluate predictive performance under varying levels of information granularity, model development was carried out using four distinct categories of input datasets:

1. Clinical variables exclusively, serving as a baseline representation of patient-specific characteristics;
2. Conventional whole-tumor radiomic features, reflecting global phenotypic descriptors extracted from the entire lesion volume;
3. Habitat-based radiomic features, capturing spatially heterogeneous subregional imaging signatures;
4. A multimodal integration of clinical parameters with radiomic features, designed to exploit the complementary strengths of clinical and imaging-derived information.

The rationale for this multi-tiered dataset construction was to investigate not only the independent predictive value of clinical and radiomic features but also the extent to which their integration could enhance model robustness and generalizability across heterogeneous patient populations [7,31].

### 2.6. Reproducibility and Robustness Assessment

To ensure reproducibility and robustness of the radiomics workflow, both segmentation reliability and feature stability were systematically evaluated. Tumor regions of interest (ROIs) were independently delineated by two radiologists with more than 10 years of experience in gynecologic imaging. Inter- and intra-observer reproducibility was assessed in 30 randomly selected cases using intraclass correlation coefficients (ICC). An ICC ≥ 0.80 was considered indicative of good reproducibility, and only features meeting this criterion were retained for further analysis. Feature extraction strictly followed Image Biomarker Standardisation Initiative (IBSI) guidelines to enhance reproducibility across studies. In addition, all images were resampled to an isotropic voxel size of $1\times 1\times 1\;{\mathrm{mm}}^{3}$ and normalized prior to feature extraction to reduce scanner- and acquisition-related variability.

To further improve model robustness, feature selection was performed using a multi-step strategy, including correlation analysis and LASSO regression with ten-fold cross-validation. Random seeds were fixed during model training to minimize stochastic variation. These procedures collectively ensured that the proposed model was reproducible and suitable for potential external validation.

### 2.7. Model Evaluation

To comprehensively assess model reliability and clinical applicability, three validation strategies were employed. First, calibration performance was evaluated using calibration curves to examine the agreement between predicted probabilities and observed outcomes and to reflect the accuracy of risk estimation. Second, decision curve analysis (DCA) was performed to quantify net clinical benefit across a range of threshold probabilities, with particular emphasis on the 0.1–0.4 interval, which is clinically relevant for preoperative decision-making in cervical cancer. Finally, a nomogram was constructed based on multivariate regression, integrating significant clinical variables with habitat-based radiomic features, to provide an intuitive tool for individualized risk prediction and to support treatment planning prior to surgery.

### 2.8. Statistical Analysis

The distribution of continuous variables was assessed using the Shapiro–Wilk test. Depending on normality, comparisons were performed using either the Student’s *t*-test or the Mann–Whitney U-test. Categorical variables were compared using the chi-square test. No significant differences were observed between the training and validation cohorts (p>0.05), confirming balanced group allocation [32,33].

All statistical analyses were conducted on the OnekeyAI platform (v3.1.8) using Python 3.7.12. Statistical modelling was performed with Statsmodels (v0.13.2), radiomic feature extraction with PyRadiomics (v3.0.1), and machine-learning algorithms with Scikit-learn (v1.0.2) [34].

## 3. Results

### 3.1. Patient Characteristics

A total of 149 patients with early-stage cervical cancer were analysed. The median age was 51 years (range, 28–70 years). Pathological assessment confirmed lymph node metastasis in 106 patients (71.14%), while the remaining 43 patients (28.86%) were node-negative. Baseline clinicopathological characteristics, including age, stromal invasion, lymphovascular space invasion (LVSI), FIGO stage, histological subtype, and treatment status, are summarized in Table 2. No significant differences were observed between the training and validation cohorts (all *p* > 0.05), indicating well-balanced distributions.

### 3.2. Univariate and Multivariate Analyses

In univariate logistic regression, FIGO stage, LVSI, and cervical stromal invasion were significantly associated with nodal metastasis (all *p* < 0.05). Multivariate analysis demonstrated that only LVSI remained an independent predictor of nodal status (OR = 1.321, 95% CI: 1.154–1.513, *p* < 0.05). These findings are consistent with previous studies highlighting LVSI as a robust prognostic factor for nodal involvement [35,36]. Detailed results of univariate and multivariate analyses are shown in Table 3. LVSI has been consistently reported as an adverse prognostic factor for nodal involvement in early-stage cervical cancer.

### 3.3. Reproducibility of Radiomic Features and Habitat Segmentation

Inter- and intra-observer reproducibility analysis demonstrated that a substantial proportion of radiomic features were robust, with 32 of the initial features achieving intraclass correlation coefficients (ICCs) ≥ 0.80. These stable features were retained for further modelling. As summarized in Table 4, tumor volumetry and first-order features exhibited the highest reproducibility (median ICCs of 0.89 and 0.91, respectively), whereas texture and GLCM features showed relatively lower but acceptable consistency. These observations align with prior radiomics reproducibility analyses [12,37]. The proportion of cases requiring arbitration remained low across all feature categories, ranging from 3.1% to 15.3%.Feature computation adhered to radiomics standardization guidance to enhance repeatability and comparability [12]; prior work has highlighted reproducibility constraints and mitigation strategies in radiomics [37].

**Figure 3:**
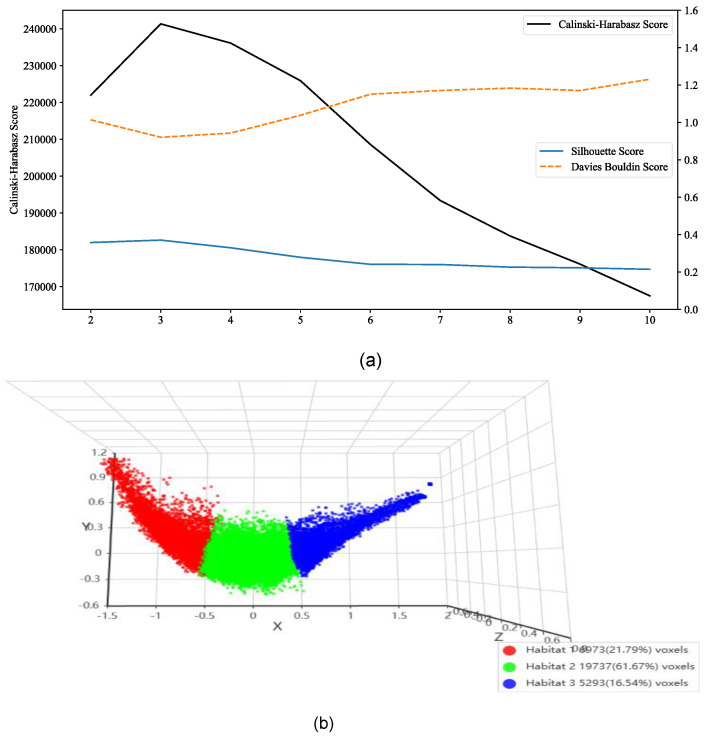
(**a**) Cluster evaluation curves showing Calinski–Harabasz, Silhouette, and Davies–Bouldin indices across κ = 2–10, with κ = 3 identified as optimal; (**b**) Three-dimensional visualization of tumor habitat segmentation at κ = 3, illustrating three distinct subregions and their voxel proportions.

To capture intratumoral spatial heterogeneity, voxel-wise k-means clustering (κ = 2–10) was performed within tumor regions. Cluster evaluation metrics, including the Calinski–Harabasz, Silhouette, and Davies–Bouldin indices, consistently identified κ = 3 as the optimal solution (Figure 3a). Based on this scheme, tumors were partitioned into three distinct subregions (habitats), accounting for 21.79%, 61.67%, and 16.54% of the total tumor voxels, respectively (Figure 3b). This habitat-based approach provided a reproducible representation of intratumoral heterogeneity, forming the basis for subsequent feature extraction and modelling [38].

### 3.4. Feature Selection

**Figure 4:**
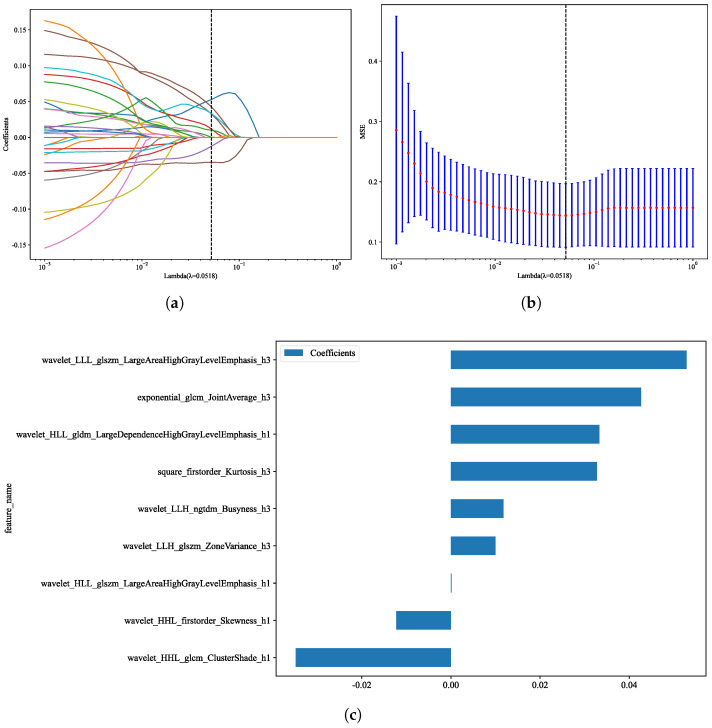
(**a**) LASSO coefficient profiles of radiomic features across a range of λ values, with the vertical dashed line indicating the optimal λ; (**b**) Ten-fold cross-validation curve showing the mean squared error (MSE) across λ values, with error bars representing standard deviation; (**c**) Radiomic features retained at the optimal λ, with corresponding coefficients.

To reduce redundancy and mitigate overfitting, least absolute shrinkage and selection operator (LASSO) regression with ten-fold cross-validation was employed [39]. As illustrated in Figure 4a, increasing values of the regularization parameter (λ) progressively shrank the coefficients of less informative features toward zero, while only the most predictive features remained non-zero. The optimal λ was identified at 0.0518, corresponding to the vertical dashed line.

Cross-validation curves further supported the selection of this λ value, which minimized the mean squared error (MSE) across folds and provided a balance between model parsimony and predictive accuracy (Figure 4b).

At the chosen λ, a subset of nine radiomic features was retained, as shown in Figure 4c. These features encompassed first-order statistics (e.g., Kurtosis, Skewness), texture descriptors derived from GLCM and GLDM, and higher-order wavelet-transformed features. Together, this compact feature set captured complementary aspects of tumor heterogeneity and was used for subsequent model construction.

### 3.5. Model Construction and Performance Evaluation

**Figure 5:**
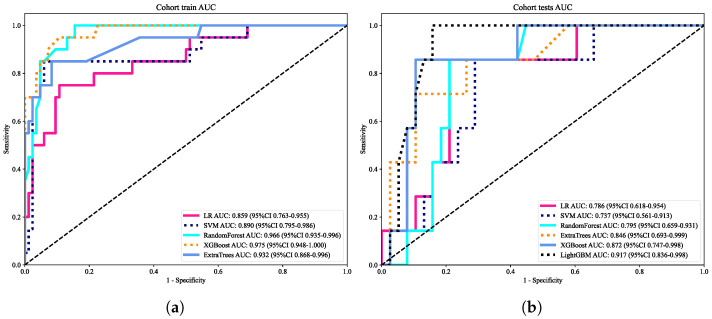
(**a**) Receiver operating characteristic (ROC) curves of five machine-learning classifiers (LR, SVM, RF, XGBoost, ExtraTrees) in the training cohort, illustrating the superior discriminative performance of ensemble-based methods; (**b**) ROC curves of the same classifiers in the independent test cohort, showing consistent robustness of ensemble models compared with LR and SVM.

The predictive performance of multiple machine learning classifiers, including logistic regression (LR), support vector machine (SVM), random forest (RF), extreme gradient boosting (XGBoost), and extra trees, was systematically evaluated. As summarized in Table 5, XGBoost and RF achieved the highest discriminative ability in the training cohort, with AUCs of 0.975 and 0.966, respectively, while maintaining robust generalization in the independent test cohort (AUC = 0.872 and 0.795, respectively). Corresponding ROC curves are presented in Figure 5a,b. Ensemble methods often provide competitive performance in radiomics classification tasks [6,40]. Highlighting consistent superiority of ensemble-based methods over conventional LR and SVM.

### 3.6. Model Comparison, Calibration, and Clinical Utility

#### 3.6.1. Model Comparison

**Figure 6:**
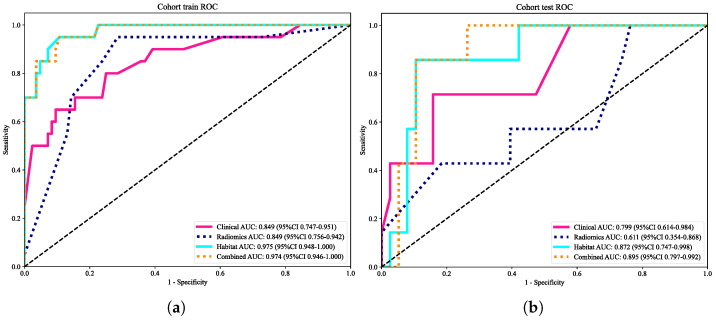
Receiver operating characteristic (ROC) curves of different models in the training cohort (**a**) and validation cohort (**b**). The combined model consistently demonstrated superior discriminative ability compared with the clinical-, radiomics-, and habitat-only models.

We constructed four models using different input feature sets: (**a**) clinical variables only, (**b**) conventional whole-tumor radiomic features, (**c**) habitat-based radiomic features, and (**d**) a combined model integrating clinical and radiomic features. As summarized in Figure 6, the habitat model significantly outperformed the conventional radiomics model in both the training (AUC = 0.975 vs. 0.849) and validation cohorts (AUC = 0.872 vs. 0.611). The combined model achieved the best overall performance, with an AUC of 0.974 in the training set and 0.895 in the validation set.

#### 3.6.2. Delong Test Comparison

**Figure 7:**
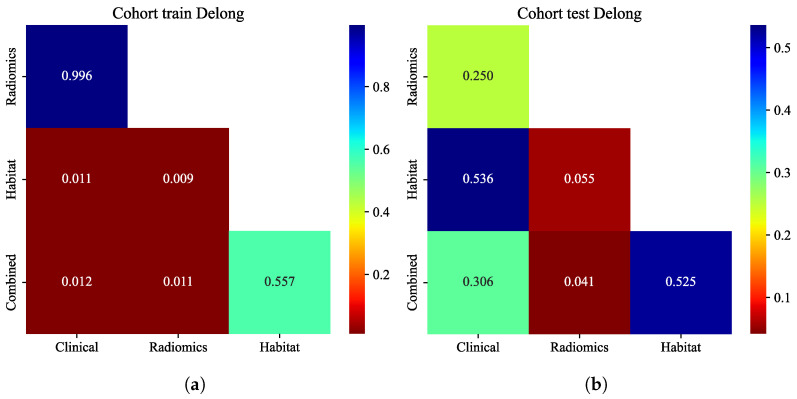
Delong test heatmaps comparing the AUCs among the clinical, radiomics, habitat, and combined models in the training cohort (**a**) and validation cohort (**b**). The combined model showed significantly higher performance than the clinical and radiomics models (*p* < 0.05), while no significant difference was observed compared with the habitat model.

Pairwise comparisons of AUCs were assessed using the DeLong approach (Figure 7a,b) [41]. In the training cohort, the combined model achieved significantly higher AUCs than the clinical and radiomics models (*p* < 0.05), whereas no significant difference was observed compared with the habitat model (*p* > 0.05). In the validation cohort, the combined model also significantly outperformed the radiomics model (*p* < 0.05), while differences relative to the clinical and habitat models were not statistically significant (*p* > 0.05). These results confirm the incremental value of incorporating habitat-derived features.

#### 3.6.3. Calibration Analysis

**Figure 8:**
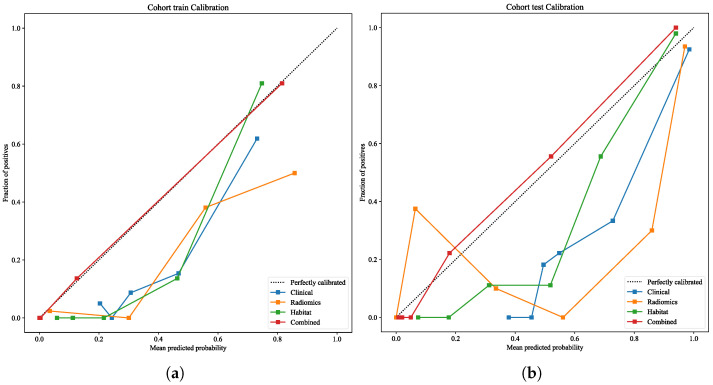
Calibration curves of the clinical, radiomics, habitat, and combined models in the training cohort (**a**) and validation cohort (**b**). The combined model achieved the best agreement between predicted probabilities and observed outcomes.

Calibration plots (Figure 8a,b) were generated following current recommendations that emphasize calibration as a critical dimension of model validity [42]. The habitat model showed moderate calibration, whereas the clinical and radiomics models deviated substantially from the reference line in the validation set, reflecting limited stability.

#### 3.6.4. Clinical Utility

**Figure 9:**
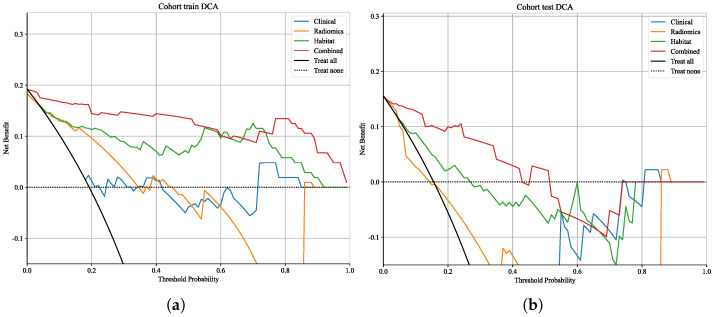
Decision curve analysis (DCA) of the clinical, radiomics, habitat, and combined models in the training cohort (**a**) and validation cohort (**b**). The combined model provided the greatest net clinical benefit within the threshold probability range of 0.1–0.4.

**Figure 10:**
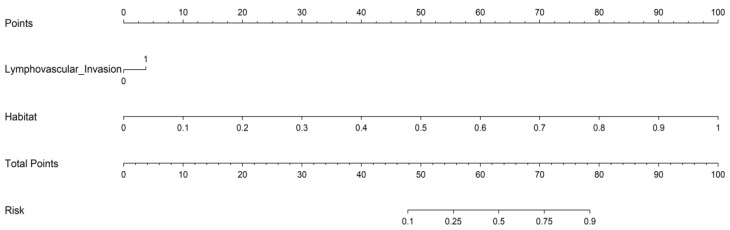
Nomogram integrating lymphovascular space invasion (LVSI) and habitat-based radiomic features for individualized prediction of lymph node metastasis in early-stage cervical cancer.The nomogram provides an intuitive clinical tool for personalized risk stratification.

Decision curve analysis (DCA, Figure 9) further confirmed the clinical utility of the combined model [43,44]. Within a clinically relevant threshold probability range (0.1–0.4), the combined model consistently yielded the greatest net benefit compared with the other models. The habitat model provided modest benefit at lower thresholds but declined rapidly at higher thresholds, while the clinical model offered only marginal benefit at higher thresholds. The radiomics model approximated or fell below the “treat-all” strategy, indicating minimal standalone clinical value. To facilitate clinical application, we developed a nomogram integrating LVSI and habitat features (Figure 10), which provides an intuitive tool for individualized prediction of lymph node metastasis risk [45,46].

## 4. Discussion

This study demonstrates that lesion-based MRI radiomics, particularly when integrated with clinicopathological variables, substantially improves the preoperative prediction of lymph node metastasis (LNM) in early-stage cervical cancer. Compared with conventional whole-tumor radiomics or clinical models alone, the proposed habitat-oriented strategy achieved superior discrimination, calibration, and clinical utility. Importantly, these advantages were consistently observed in the internal validation cohort, underscoring the robustness and reliability of the proposed approach [47].

Previous studies have explored MRI-based radiomics for predicting nodal status in cervical cancer; however, most relied on features extracted from the entire tumor volume, potentially masking biologically meaningful intratumoral heterogeneity [47]. Emerging evidence suggests that subregional or habitat-based analysis can better characterize spatial heterogeneity and improve predictive performance [38,48]. Our findings extend this concept by systematically applying voxel-level clustering to derive intratumoral habitats, demonstrating that tumor-intrinsic imaging heterogeneity conveys clinically relevant information associated with metastatic potential [38,48,49].

Compared with previous MRI-based radiomics studies for predicting lymph node metastasis in cervical cancer, including the work by Liu et al. and the meta-analysis by Li et al. [50,51], the present study demonstrates several important methodological and clinical advances. Most prior studies relied on conventional whole-tumor radiomic features, which implicitly assume tumor homogeneity and may obscure biologically meaningful spatial variation. In contrast, we introduced a habitat-based radiomics framework that explicitly models intratumoral heterogeneity by voxel-wise clustering of DWI/ADC images into distinct subregions. This approach enables the extraction of subregional imaging signatures that reflect diverse tumor microenvironments, thereby providing complementary information beyond global radiomic descriptors. As a result, the habitat-based model significantly outperformed conventional radiomics in the validation cohort, and further integration with clinical variables yielded the highest discriminative performance, robust calibration, and superior clinical net benefit. These findings directly address the heterogeneity and limited clinical utility highlighted in previous systematic reviews and support habitat radiomics as a more biologically informed and clinically translatable strategy for preoperative LNM prediction in early-stage cervical cancer.

The improved performance of habitat-based radiomics may reflect underlying biological variations within tumors. Distinct intratumoral subregions are likely associated with differences in cellular density, hypoxia, necrosis, and angiogenesis—pathophysiological processes known to influence tumor aggressiveness and metastatic behavior [49]. By quantitatively capturing such heterogeneity, the habitat-based model provides complementary information beyond conventional size- or morphology-based imaging metrics. From a clinical perspective, accurate preoperative assessment of nodal status is essential for individualized treatment planning. To facilitate clinical translation, we developed a nomogram integrating lymphovascular space invasion (LVSI) and habitat-based radiomic features, and evaluated its clinical utility using decision curve analysis (DCA), in accordance with current methodological recommendations [52].

Several methodological strengths enhance the credibility of our results. First, all tumor regions of interest were manually delineated by experienced radiologists, with intra- and inter-observer reproducibility systematically assessed using intraclass correlation coefficients, and radiomic feature extraction strictly adhered to IBSI guidelines [12]. Second, a structured and multi-step feature selection pipeline—including univariate screening, correlation analysis, and LASSO regression—was employed to reduce redundancy and minimize overfitting. Third, model robustness was evaluated across multiple machine learning classifiers, and performance stability was confirmed through internal validation [47]. Calibration analysis emphasized probability reliability as a core component of predictive model validity, while DCA provided insight into potential clinical benefit [42,52].

Nevertheless, several limitations of this study should be acknowledged. First, this was a retrospective, single-center study with a relatively modest sample size, which may introduce selection bias and limit generalizability. Second, although MRI acquisition parameters were standardized within a single scanner and protocol—helping to reduce imaging-related variability—model performance may be affected when applied across different scanners or imaging protocols. External validation using multicenter datasets is therefore necessary to confirm the robustness and general applicability of the proposed model. Third, while reproducibility of radiomic features was acceptable, texture features remain inherently sensitive to segmentation variability, highlighting the need for automated or semi-automated segmentation methods in future studies [53,54]. In addition, habitat construction in this work was based primarily on DWI and ADC images; incorporation of multiparametric MRI sequences may further enrich biological characterization [49]. Future work should focus on prospective, multicenter validation with larger cohorts to strengthen the clinical evidence base. Integration of radiogenomic data and longitudinal imaging may also improve biological interpretability and predictive performance, ultimately supporting broader clinical implementation [5].

## 5. Conclusions

In conclusion, this study demonstrates that lesion-based MRI habitat radiomics, particularly when integrated with clinicopathological variables, provides a robust and non-invasive approach for preoperative prediction of lymph node metastasis in early-stage cervical cancer. By capturing intratumoral heterogeneity that is overlooked by conventional whole-tumor radiomics, the proposed combined model achieved improved discriminative performance, reliable calibration, and meaningful clinical utility.

Despite the encouraging results observed in internal validation, further validation in larger, prospective, multicenter cohorts is warranted to confirm the generalizability and clinical applicability of the model across different imaging protocols and patient populations. Nevertheless, this habitat-based radiomics framework represents a promising step toward precision-guided preoperative risk stratification and individualized management of cervical cancer, and provides a solid foundation for future translational and clinical studies [5,12,42,47]. 

## Figures and Tables

**Table 1:** The baseline characteristics, clinicopathological features, and treatment methods of the patients (n = 149). The table shows age, HPV infection status, lymph node metastasis, lymphovascular invasion, depth of cervical invasion, FIGO stage, and chemotherapy and radiotherapy. Data are presented as mean ± standard deviation, median (range), frequency (percentage), and 95% confidence interval.

Characteristic	Category/Statistic	Value (n = 149)	Percentage	95% Confidence Interval
Age	Mean ± SD	52.06 ± 9.66		(50.51, 53.61)
	Median (Range)	51 (28–70)		
HPV Status	Positive	102	68.46%	(60.99%, 75.93%)
	Negative	12	8.05%	(3.68%, 12.42%)
	Not reported	35	23.49%	(16.69%, 30.29%)
Lymph Node Metastasis	Absent	124	83.22%	(77.22%, 89.22%)
	Present	25	16.78%	(10.78%, 22.78%)
Lymphovascular Invasion	Absent	106	71.14%	(63.87%, 78.41%)
	Present	43	28.86%	(21.59%, 36.13%)
Depth of Cervical Invasion (cm)	Mean ± SD	0.62 ± 0.27		(0.58, 0.66)
	Median (Range)	0.6 (0.1–1.0)		
FIGO Stage	I (A/B)	92	61.74%	(53.94%, 69.54%)
	II (A/B)	52	34.90%	(27.24%, 42.56%)
	III/IV	5	3.36%	(0.46%, 6.26%)
Treatment	Chemotherapy (Yes)	132	88.59%	(83.49%, 93.69%)
	Radiotherapy (Yes)	101	67.79%	(60.28%, 75.30%)

**Table 2:** The baseline characteristics, clinicopathological features and treatment methods of the patients were summarized (n = 149). This table compares the baseline clinical characteristics of all patients (ALL set), the test set, and the training set. Variables include age, cervical stromal invasion, lymphovascular invasion, clinical stage, chemotherapy, and radiotherapy. No significant differences were observed among the three groups (*p* > 0.05), indicating good comparability between the training and test sets.

Feature		All	Test	Train	*p*-Value
Age		52.06 ± 9.66	51.02 ± 8.13	52.51 ± 10.26	0.39
Cervical_Stromal_Invasion		0.62 ± 0.27	0.63 ± 0.26	0.62 ± 0.27	0.83
Lymphovascular_Invasion					0.558
	Absent	106 (71.14)	34 (75.56)	72 (69.23)	
	Present	43 (28.86)	11 (24.44)	32 (30.77)	
Stage					0.879
	*I* (A/B)	92 (61.74)	28 (62.22)	64 (61.54)	
	II (A/B)	52 (34.90)	16 (35.56)	36 (34.62)	
	III/IV	5 (3.36)	1 (2.22)	4 (3.85)	
Chemotherapy					0.359
	No	17 (11.41)	3 (6.67)	14 (13.46)	
	Yes	132 (88.59)	42 (93.33)	90 (86.54)	
Radiotherapy					0.253
	No	48 (32.21)	11 (24.44)	37 (35.58)	
	Yes	101 (67.79)	34 (75.56)	67 (64.42)	

**Table 3:** Univariate and multivariate logistic regression analyses of clinicopathological predictors for lymph node metastasis. OR = odds ratio; CI = confidence interval; UNI = univariate analysis; MULTI = multivariate analysis. Variables with *p* < 0.05 in univariate analysis were entered into multivariate analysis. Bold values indicate statistical significance (*p* < 0.05).

Feature_Name	OR_UNI	95% CI_UNI	*p*_Value_UNI	OR_MULTI	95% CI_MULTI	*p*_Value_MULTI
Age	1.003	0.9970–1.0100	**0.401**			
Chemotherapy	1.059	0.8760–1.2800	**0.618**			
Radiotherapy	1.093	0.9550–1.2500	**0.276**			
Stage	1.146	1.0240–1.2810	<**0.05**	1.105	0.9900–1.2340	**0.136**
Lymphovascular_Invasion	1.362	1.1950–1.5530	<**0.05**	1.321	1.1540–1.5130	<**0.05**
Cervical_Stromal_Invasion	1.367	1.0800–1.7320	<**0.05**	1.125	0.8840–1.4320	**0.419**

**Table 4:** Inter-observer reproducibility of radiomic features assessed by intraclass correlation coefficients (ICC).

Feature Category	Median ICC	IQR	95% CI	Cases Requiring Arbitration (%)
Tumor Volumetry	0.89	0.86–0.92	0.83–0.93	5.8
First-Order	0.91	0.86–0.94	0.85–0.95	3.1
Texture Features	0.79	0.72–0.85	0.74–0.84	12.6
GLCM Features	0.76	0.68–0.82	0.71–0.81	15.3
Overall	0.82	0.77–0.87	0.77–0.87	11.4

**Table 5:** Performance of different machine-learning classifiers in predicting lymph, node metastasis across the training and test cohorts. Metrics include accuracy, AUC, 95% confidence interval (CI), sensitivity, specificity, F1 score, and net benefit.

Model Name	Accuracy	AUC	95% CI	Sensitivity	Specificity	PPV	NPV	Cohort
LR	0.865	0.859	0.763–0.955	0.750	0.893	0.625	0.937	train
LR	0.800	0.786	0.618–0.954	0.857	0.789	0.429	0.968	test
SVM	0.923	0.890	0.795–0.986	0.850	0.940	0.773	0.963	train
SVM	0.733	0.737	0.561–0.913	0.857	0.711	0.353	0.964	test
Random Forest	0.875	0.966	0.935–0.996	1.000	0.845	0.606	1.000	train
Random Forest	0.800	0.795	0.659–0.931	0.857	0.789	0.429	0.968	test
XGBoost	0.904	0.975	0.948–1.000	0.950	0.893	0.679	0.987	train
XGBoost	0.889	0.872	0.747–0.998	0.857	0.895	0.600	0.971	test
Extra Trees	0.904	0.932	0.868–0.996	0.850	0.917	0.708	0.962	train
Extra Trees	0.867	0.847	0.693–0.999	0.714	0.895	0.556	0.944	test

## Data Availability

Data are available on request to the authors.

## Abbreviations

The following abbreviations are used in this manuscript:

LNM	lymph node metastasis
ADC	Apparent Diffusion Coefficient
ROC	Receiver Operating Characteristic
AUC	Area under Curve
DCA	Decision Curve Analysis
LASSO	Least Absolute Shrinkage and Selection Operator
DWI	Diffusion-weighted Image
$T_{2}$WI	$T_{2}$-weighted and Diffusion-weighted Images
ROI	Region of Interest
GLCM	Gray-level Co-occurrence Matrix
GLRLM	Gray-level Run-length Matrix
GLSZM	Gray-level Size Zone Matrix
ICC	Intraclass Correlation Coefficient
LR	Logistic Regression
SVM	Support Vector Machine
RF	Random Forest
XGBoost	Extreme Gradient Boosting
LVSI	Lymphovascular Space Invasion
OR	Odds Ratio
CI	Confidence Interval
UNI	Univariate Analysis
MULTI	Multivariate Analysis
MSE	Mean Squared Error
